# Clinical and Microbiological Profile of Hospital-Acquired and Ventilator-Associated Pneumonia in Critically Ill Patients: A Retrospective Observational Study

**DOI:** 10.3390/antibiotics15020232

**Published:** 2026-02-22

**Authors:** Mihnea Miron, Anca Irina Ristescu, Mihaela Blaj, Diana Gabriela Iosep, Alexandru-Florinel Oancea, Gabriel Iosep, Radu Crișan-Dabija, Daniela Diculencu, Costin Damian, Mihaela Cătălina Luca

**Affiliations:** 1 Grigore T. Popa University of Medicine and Pharmacy, 700115 Iași, Romania; miron_mihnea@d.umfiasi.ro (M.M.); diana.iosep@umfiasi.ro (D.G.I.); alexandru.oancea@umfiasi.ro (A.-F.O.); radu.dabija@umfiasi.ro (R.C.-D.); 2 Department of Anesthesia and Intensive Care, Faculty of Medicine, Grigore T. Popa University of Medicine and Pharmacy, 700115 Iași, Romania; mihaela.blaj@umfiasi.ro; 3 Anesthesiology and Intensive Care Unit, Regional Institute of Oncology, 700483 Iași, Romania; 4 Anesthesiology and Intensive Care Unit, “Sf. Spiridon” County University Emergency Hospital, 700111 Iași, Romania; 5 Anesthesiology and Intensive Care Unit, Clinical Hospital of Pneumology, 700115 Iași, Romania; gabriel.iosep@pneumo-iasi.ro; 6 Pulmonology Department, Clinical Hospital of Pneumology, 700115 Iași, Romania; 7 Medical Analysis Laboratory, Clinical Hospital of Pneumology, 700115 Iași, Romania; 8 Clinic of Infectious Diseases, “Sf. Parascheva” Clinical Hospital of Infectious Diseases, 700116 Iași, Romania

**Keywords:** hospital-acquired pneumonia, ventilator-associated pneumonia, antimicrobial resistance, ESKAPE pathogens, acute kidney injury, SOFA-2 score

## Abstract

**Background/Objectives**: Severe respiratory infections remain a major cause of morbidity and mortality in critically ill patients admitted to an intensive care unit (ICU), particularly in the context of increasing antimicrobial resistance (AMR). This study aimed to describe the clinical, microbiological and resistance profiles of ICU patients diagnosed with hospital-acquired or ventilator-associated pneumonia. **Methods**: We conducted a retrospective, single-center observational study including adult ICU patients admitted between January and December 2025, with clinically significant positive endotracheal aspirates. Clinical severity scores (APACHE II, SOFA, SOFA-2), inflammatory biomarkers (neutrophil-to-lymphocyte ratio—NLR, platelets-to-lymphocyte ratio—PLR, C-reactive protein—CRP), microbiological findings, antimicrobial resistance patterns and ICU-related outcomes were analyzed. **Results**: Out of the 606 endotracheal aspirates collected, 76 (12.5%) were culture-positive and 62 (10.2%) patients met the final inclusion criteria. Ventilator-associated pneumonia accounted for 90% of infections, 25 episodes (44.6%) being classified as early-onset and 31 cases (55.4%) as late-onset, without significant differences in bacterial distribution between the two subtypes. In total, 85.5% of infections were monomicrobial, with Gram-negative bacteria representing 76% of isolates. *Acinetobacter baumannii* and *Pseudomonas aeruginosa* were the most frequently isolated pathogens, with high resistance rates. Acute kidney injury occurred in 25.8% of patients and was associated with higher APACHE II, SOFA, and SOFA-2 scores. **Conclusions**: Severe respiratory infections in critically ill patients were predominantly caused by Gram-negative, frequently drug-resistant pathogens and were associated with high disease severity and poor outcomes. These findings provide insight into the local epidemiology and antimicrobial resistance patterns of severe respiratory infections in critically ill patients.

## 1. Introduction

Severe respiratory infections (RTIs) represent a significant cause of both morbidity and mortality among critically ill patients admitted to intensive care units (ICUs) worldwide [1]. Amid these infections, the clinical spectrum of nosocomial pneumonia, including hospital-acquired pneumonia (HAP) and ventilator-associated pneumonia (VAP), poses significant concerns regarding not only unfavorable clinical outcomes, but also increased healthcare costs with prolonged ICU and hospital length of stay (LOS) [2,3]. Despite recent advances in critical care management and antimicrobial therapy, lower respiratory tract infections (LRTIs) persist as a considerable burden in mechanically ventilated patients with advanced respiratory diseases [4].

Severe RTIs requiring ICU admission can be determined by either community-acquired or hospital-acquired pathogens. There is a large geographic variability across countries, regions and even between individual ICUs concerning both microbial etiology and antimicrobial resistance patterns. Whereas community-acquired pneumonia (CAP) is most often caused by typical bacterial pathogens, namely *Streptococcus pneumoniae*, *Haemophilus influenzae* and *Staphylococcus aureus*, along with atypical agents, HAP and VAP are predominantly caused by opportunistic and frequently multidrug-resistant (MDR) pathogens [5,6]. Therefore, Gram-negative bacteria including *Acinetobacter baumannii*, *Pseudomonas aeruginosa*, *Klebsiella pneumoniae* and *Escherichia coli*, together with Gram-positive microorganisms, including *Staphylococcus aureus* and *Enterococcus* spp. constitute the most commonly reported causative agents of RTIs in patients who require ICU admission and, more importantly, invasive mechanical ventilation [7,8,9]. Notably, many of the pathogens previously mentioned belong to the ESKAPE group (*Enterococcus faecium*, *Staphylococcus aureus*, *Klebsiella pneumoniae*, *Acinetobacter baumannii*, *Pseudomonas aeruginosa* and *Enterobacter* spp.), which is widely recognized as a significant cause of nosocomial infections in critically ill patients, as well as a major driver of antimicrobial resistance in ICU [10].

Lately, antimicrobial resistance (AMR) has been considered a major global public health threat, with ICUs serving as high-risk settings for both emergence and dissemination of MDR agents. Moreover, several intrinsic and acquired mechanisms have been described which limit the efficacy of currently available antimicrobials, with a significant impact on ICU mortality rate, as a primary outcome [11]. Thus, the six most common mechanisms cited in the recent literature are enzymatic drug inactivation, target site modification, decreased drug uptake, efflux pump overexpression, biofilm formation with acquired tolerance and horizontal gene transfer (HGT), collectively leading to limited therapeutic options in ICU-admitted patients [10,12,13].

In addition to microbiological characterization, evaluating and integrating disease severity through clinical scores and biological parameters are crucial for guiding treatment decisions, as well as for predicting outcomes in critically ill patients with severe RTIs. Therefore, clinical scoring systems, as the Acute Physiology and Chronic Health Evaluation II (APACHE II) and also Sequential Organ Failure Assessment (SOFA) scores, including the recently updated SOFA-2 version, are widely used tools to quantify illness severity and organ dysfunction in critical patients [14,15]. It is also noteworthy that routine laboratory-derived inflammatory biomarkers, including neutrophil-to-lymphocyte ratio (NLR) and platelet-to-lymphocyte ratio (PLR), have increasingly been recognized as useful indicators of systemic inflammation, which represents the cornerstone in the development and progression of critical illness [16,17].

The SOFA-2 score was recently developed to refine organ dysfunction assessment in critically ill patients, including contemporary organ support techniques and revised thresholds which better reflect current intensive care management. Moreover, aiming to improve both descriptive and prognostic validity compared to the original SOFA score, SOFA-2 preserves the evaluation of the six classical organ systems (central nervous, respiratory, cardiovascular, hepatic, renal and coagulation) and integrates updated scoring criteria aligned with contemporary ICU practice [14,18,19].

Beyond clinical severity scoring, routinely available inflammatory ratios may further optimize risk stratification in critical patients. The neutrophil-to-lymphocyte ratio (NLR) is a useful biomarker that supports diagnostic evaluation, offers prognostic information and may assist in monitoring treatment response. The NLR has been associated with immune dysregulation and adverse outcomes in sepsis and severe RTIs, whereas the PLR reflects platelet-driven inflammatory and prothrombotic pathways in infection-related organ dysfunction [16,17,20,21].

Hence, combining clinical severity scores with microbiological data, together with easily accessible inflammatory ratios, may provide a comprehensive insight into the complex interaction between the immune host response, microbial virulence and patient outcomes.

Currently, despite the extensive scientific literature addressing hospital-acquired and ventilator-associated pneumonia, data from Eastern European ICUs remain underreported. This gap is clinically relevant since Romania, among other neighboring countries, reports a high prevalence of MDR pathogens, hindering empirical antibiotic selection and altering patient outcomes. Therefore, region-specific epidemiological data integrating microbiological resistance patterns with updated severity assessment tools are needed to optimize antimicrobial stewardship and risk stratification strategies in critically ill patients.

This study’s primary objective was to characterize the clinical features, microbiological profile and antimicrobial resistance patterns of HAP and VAP in patients admitted to a tertiary-care ICU in Romania. Secondary objectives included comparative analyses depending on the type of infection and microbiological resistance profile.

## 2. Results

### 2.1. Study Flowchart and Baseline Characteristics

During the study period, a total of 606 endotracheal aspirates were collected from patients admitted to the Intensive Care Unit of the Clinical Hospital of Pneumology of Iași, Romania. Among these, 76 aspirates (12.5%) yielded positive microbiological cultures. However, 14 positive aspirates were excluded from further analysis due to insufficient clinical and microbiological data, as well as for extreme disease severity at the moment of ICU admission, with death occurring within the first 24 h after admission.

The final study cohort consisted of 62 patients with positive endotracheal aspirate cultures, each contributing a single infectious episode. Of the 62 enrolled patients, 9.7% (*n* = 6) were diagnosed with hospital-acquired pneumonia (HAP) and 90.3% (*n* = 56) were diagnosed with ventilator-associated pneumonia (VAP). Among VAP cases, 44.6% *(n* = 25) were classified as early-onset VAP, whereas 55.4% (*n* = 31) were late-onset VAP.

**Figure 1:**
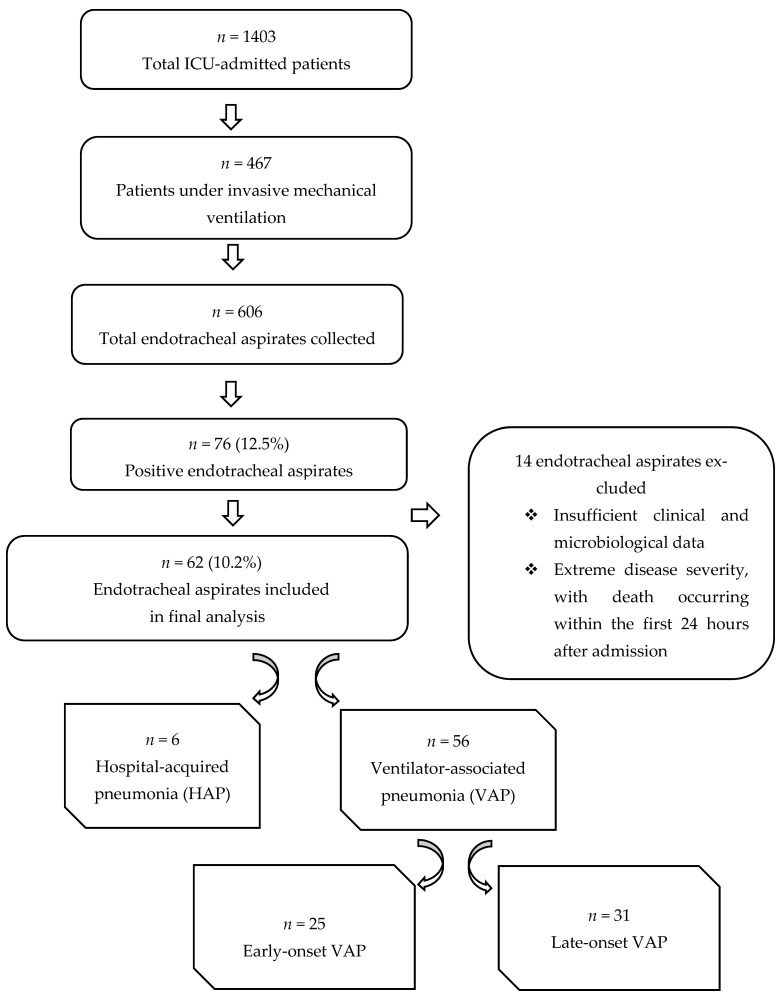
Study flowchart illustrating the selection of patients with positive endotracheal aspirate cultures included in the final analysis.

The study flowchart is illustrated in Figure 1.

Baseline demographic data, clinical severity scores, inflammatory biomarkers at ICU admission and ICU-related outcomes are summarized in Table 1.

### 2.2. Microbiological Profile

Microbiological analysis revealed the presence of both monomicrobial (85.5%, *n* = 53) and polymicrobial (14.5%, *n* = 9) respiratory infection episodes among the 62 cases included in the final cohort.

**Figure 2:**
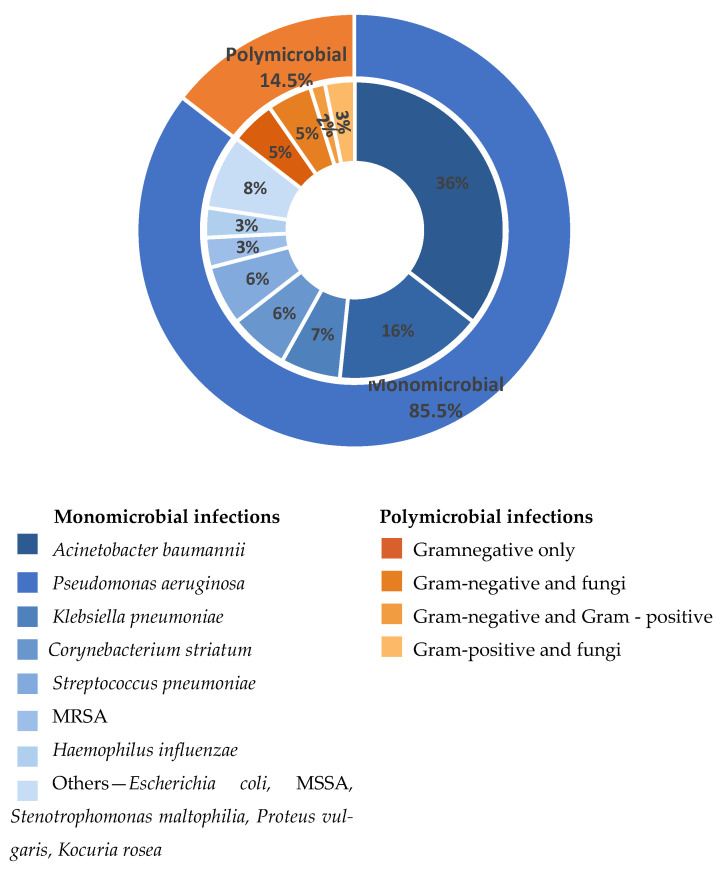
Distribution of monomicrobial and polymicrobial respiratory infections and associated microbiological findings.

An overview of monomicrobial and polymicrobial episodes and their associated microbiological findings is summarized in Figure 2.

The outer ring illustrates the overall proportion of monomicrobial and polymicrobial infections. The inner ring reflects the distribution of causative agents identified in monomicrobial infections (blue shades) and the main polymicrobial associations (orange shades). Percentages displayed in the inner ring are calculated relative to the total number of monomicrobial infections and the total number of polymicrobial infectious episodes, respectively. “Others” includes less frequently isolated pathogens, each identified in a single case, and grouped for clarity: MSSA—methicillin-susceptible *Staphylococcus aureus*; MRSA—methicillin-resistant *Staphylococcus aureus*.

#### 2.2.1. Monomicrobial Respiratory Infections

Out of all 53 monomicrobial respiratory infection episodes, 77% (*n* = 41) were determined by Gram-negative bacteria. Pathogens belonging to the ESKAPE group were frequently identified, with *Acinetobacter baumannii* being the most common causative agent, revealed in 41.5% cases (*n* = 22), followed by *Pseudomonas aeruginosa* in 10 cases (18.9%). Other ESKAPE-related pathogens included *Klebsiella pneumoniae*, identified in 7.5% of cases (*n* = 4).

Additional Gram-negative bacteria were isolated less frequently and included *Escherichia coli*, *Stenotrophomonas maltophilia* and *Proteus vulgaris*, each corresponding to a single infectious episode.

Gram-positive bacteria were less commonly isolated. *Streptococcus pneumoniae* and *Corynebacterium striatum* were each identified in 7.5% of monomicrobial respiratory infectious episodes (*n* = 4), whereas *Staphylococcus aureus*, including both methicillin-resistant (MRSA) and methicillin-susceptible (MSSA) strains, accounted for 5.7% of isolates (*n* = 3). *Haemophilus influenzae* was identified in 3.8% of cases (*n* = 2), while *Kocuria rosea* was isolated in 1.9% of cases (*n* = 1).

#### 2.2.2. Polymicrobial Respiratory Infections

Regarding the 9 polymicrobial respiratory infection episodes, mixed infections involving Gram-negative bacteria, Gram-positive bacteria and/or fungal species were observed: one case of *Acinetobacter baumannii* with *Candida* spp., one case of *Corynebacterium striatum* with *Candida albicans*, one episode with *Corynebacterium striatum* and *Acinetobacter baumannii*, one case of *Escherichia coli* and *Acinetobacter baumannii*, one case of *Enterococcus durans* and *Candida glabrata*, one case involving *Pseudomonas aeruginosa* and *Candida glabrata*, one case of *Serratia marcescens* and *Candida krusei* and two episodes consisting of *Pseudomonas aeruginosa* and *Acinetobacter baumannii* association. All fungal species and enterococci identified in polymicrobial cultures were interpreted as colonizing organisms rather than primary pulmonary pathogens and were reported descriptively.

Overall, polymicrobial infections accounted for 14.5% of all respiratory infection episodes included in our study and were mainly composed of dual microbial associations, as previously presented in Figure 2.

### 2.3. Antimicrobial Susceptibility Patterns

Antimicrobial susceptibility analysis was performed at the bacterial isolate level and included all isolates identified from both monomicrobial and polymicrobial episodes.

Among all *Acinetobacter baumannii* isolates (*n* = 27), resistance rates were particularly elevated, with six strains being classified as MDR, 15 strains as XDR and three strains as PDR, respectively. Only three isolates exhibited resistance patterns that did not fulfill the criteria for multidrug resistance, being reported as non-MDR.

**Figure 3:**
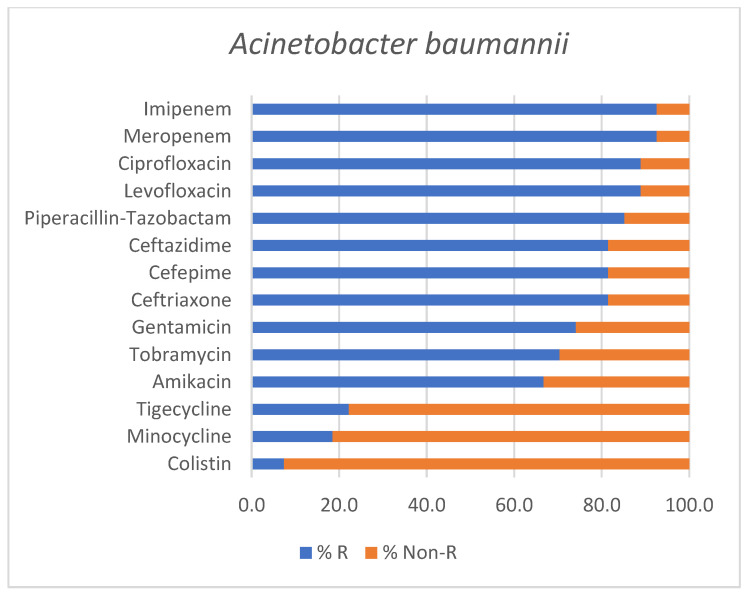
Antimicrobial susceptibility profile of *Acinetobacter baumannii* isolates identified in positive endotracheal aspirates. Bars represent the percentage of resistant (% R) and non-Resistant (% Non-R) strains for each tested antimicrobial agent.

The antimicrobial susceptibility profile of *Acinetobacter baumannii* isolates is illustrated in Figure 3.

Similarly, *Pseudomonas aeruginosa* isolates (*n* = 13) showed advanced resistance profiles, with four MDR strains, six XDR strains, and one PDR strain, while two isolates were reported as non-MDR.

**Figure 4:**
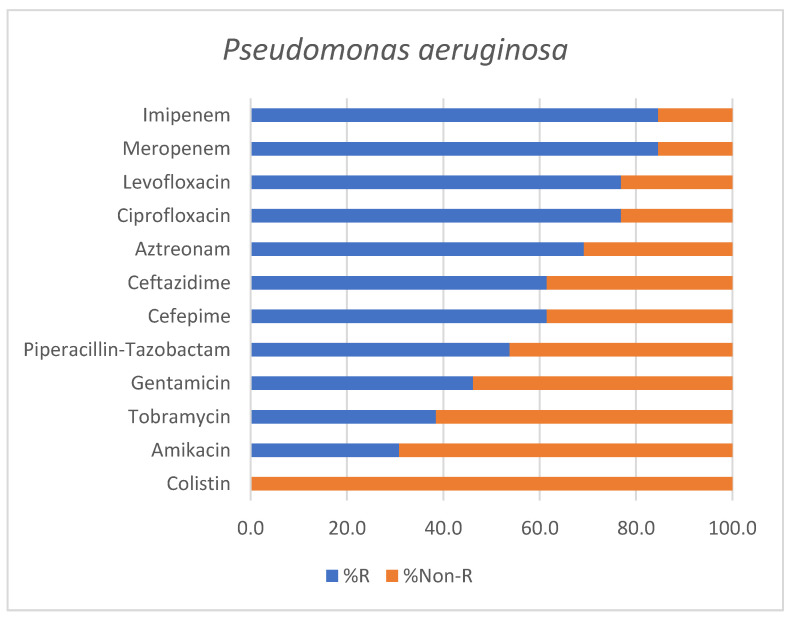
Antimicrobial susceptibility profile of *Pseudomonas aeruginosa* isolates identified in positive endotracheal aspirates. Bars represent the percentage of resistant (% R) and non-Resistant (% Non-R) strains for each tested antimicrobial agent.

The antimicrobial susceptibility profile of *Pseudomonas aeruginosa* isolates is shown in Figure 4.

**Figure 5:**
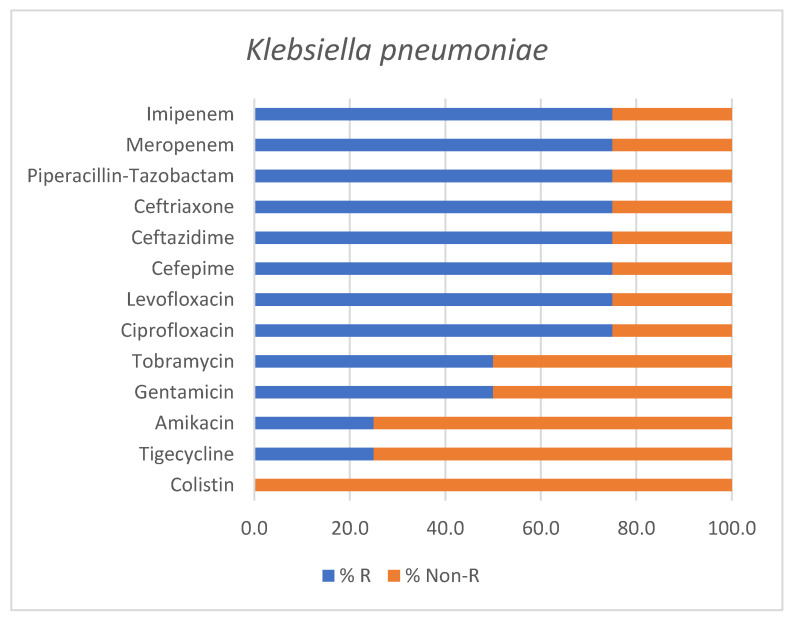
Antimicrobial susceptibility profile of *Klebsiella pneumoniae* isolates identified in positive endotracheal aspirates. Bars represent the percentage of resistant (% R) and non-Resistant (% Non-R) strains for each tested antimicrobial agent.

Out of four *Klebsiella pneumoniae* isolates, two strains were classified as PDR, one as XDR and only one as non-MDR. Although based on a limited number of isolates, the antimicrobial susceptibility profile of *Klebsiella pneumoniae* is outlined in Figure 5.

In addition to the pathogen-specific susceptibility profiles presented above, Table 2 summarizes the classification of all bacterial isolates according to antimicrobial resistance categories (MDR, XDR, PDR).

**Figure 6:**
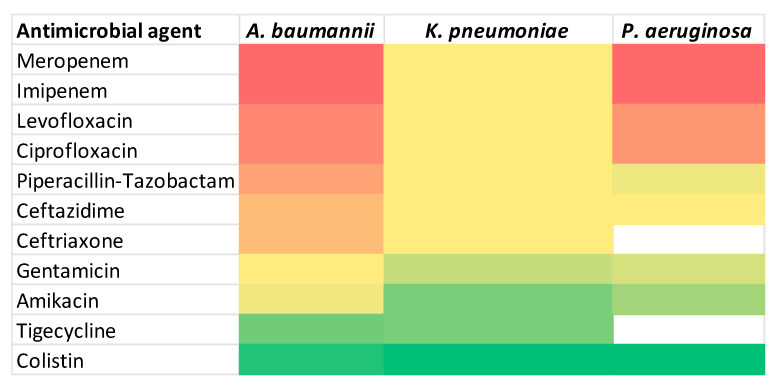
Heat map of antimicrobial resistance. Color intensity represents increasing resistance rates, ranging from green (lower resistance) to red (higher resistance). White cells indicate antibiotics that were not tested for a given pathogen.

To facilitate visual comparison of antimicrobial resistance profiles, a heat map representation of the susceptibility data is provided in Figure 6.

### 2.4. Antimicrobial Therapy

Empirical antibiotic therapy was initiated in all patients at the time of clinical diagnosis of infection. The selection of empirical antimicrobial regimens was established according to multiple factors: local epidemiological data, type of hospital-acquired infection (non ICU-aquired *versus* ICU-acquired infection), and the severity of the disease (severe infection, sepsis, septic shock). The most frequently used regimens are listed in Table 3.

Empirical antimicrobial therapy was categorized based on the initial therapeutic regimen. In cases of combination therapy, regimens were analyzed as a single treatment episode to avoid double counting. Antibiotic exposure was analyzed descriptively due to the retrospective design of the study.

### 2.5. Associations Between Antimicrobial Resistance Patterns and Clinical Profile

#### 2.5.1. Clinical Data of Patients with MDR and Non-MDR RTIs

We performed a comparative analysis between MDR and non-MDR groups. No statistically significant differences were observed between patients with MDR and non-MDR infections in terms of age, disease severity at ICU admission time (APACHE II score), organ dysfunction at the time of infection diagnosis (SOFA and SOFA-2 scores), inflammatory biomarkers (NLR, PLR, CRP), ICU LOS or duration of invasive mechanical ventilation (all *p* > 0.05). Clinical characteristics and ICU-related outcomes between MDR and non-MDR infections are presented in Table 4.

#### 2.5.2. Comparison Between Early- and Late-Onset VAP

Among all 56 patients who developed VAP, late-onset subtype predominated. Although the frequency of MDR strains was similar in early- and late-onset VAP (*p* = 1.00), the latter was associated with a significantly longer ICU LOS (median [IQR]: 14 days [10.5–21.5] vs. 6 days [4–11], *p* < 0.001) and prolonged duration of invasive mechanical ventilation (median [IQR]: 336 h [225.5–456.0] vs. 144 h [96.0–264.0], *p* < 0.001). No significant differences were observed in terms of major bacterial categories between early- and late-onset VAP, as described in Supplementary Table S1.

#### 2.5.3. Comparison Between Monomicrobial and Polymicrobial Infections

A comparative analysis between clinical parameters and ICU-related outcomes in patients with monomicrobial *versus* polymicrobial infections was performed. Patients with polymicrobial episodes exhibited longer hospital LOS (median [IQR]: 26 days [16.0–32.0] vs. 14 days [7.0–23.0] in monomicrobial group, *p* = 0.044). No significant differences were identified between the two groups with respect to disease severity scores (APACHE II, SOFA-2, SOFA), ICU LOS, duration of mechanical ventilation and development of acute kidney injury (AKI). Table 5 summarizes the comparison of clinical characteristics, disease severity scores and ICU-related outcomes between monomicrobial and polymicrobial infections.

Patients who developed AKI had significantly higher APACHE II scores at ICU admission compared to those without AKI (median [IQR]: 26.0 [23.0–31.5] vs. median 22.5 [20.0–24.0], *p* = 0.002). Similarly, SOFA and SOFA-2 scores at the time of infection diagnosis were significantly higher in the AKI group (SOFA score: median 10.0 vs. 7.0, *p* < 0.001; SOFA-2 score: median 8.5 vs. 6.0, *p* = 0.003). Acute kidney injury was neither associated with MDR infections (*p* = 1.00) nor with mortality rate (100% vs. 76.1%, *p* = 0.052). A detailed comparison of disease severity scores and ICU-related outcomes between patients with and without AKI is presented in Supplementary Table S2.

Also, exploratory correlation analyses identified several statistically significant and biologically plausible associations, as summarized in Supplementary Table S3. Thus, APACHE II score at ICU admission demonstrated a moderate positive association with SOFA score (Spearman’s *ρ* 0.34, *p* = 0.006) and SOFA-2 score (Spearman’s *ρ* 0.29, *p* = 0.023) at the time of infection diagnosis. Also, SOFA-2 score indicated a weak negative correlation with the duration of invasive mechanical ventilation (Spearman’s *ρ* −0.26, *p* = 0.037).

## 3. Discussion

The present retrospective observational study provides a systematic overview of both clinical and microbiological profiles of critically ill patients diagnosed with severe RTIs (HAP and VAP), who required admission to a tertiary-care intensive care unit. Our findings confirm the predominance of Gram-negative pathogens, especially microorganisms belonging to the ESKAPE group, and highlight the significant impact of antimicrobial resistance in this vulnerable population. Recent observational studies and reviews have consistently identified *Acinetobacter baumannii*, *Pseudomonas aeruginosa* and *Klebsiella pneumoniae* as major causative agents of MDR respiratory infections, especially in mechanically ventilated patients and in settings with high antibiotic pressure [10,22,23,24,25]. Moreover, growing evidence indicates that these agents represent major drivers for difficult-to-treat infections, prolonged ICU LOS and increased mortality, given their rapid resistance acquisition and limited treatment options [26].

Given the predominance of MDR Gram-negative pathogens, interpreting microorganisms identified in polymicrobial cultures requires careful clinical judgment. Thus, the detection of all *Candida* species in respiratory specimens was interpreted as colonization rather than true pulmonary infection, in line with current clinical evidence [27]. Similarly, the pathogenic role of *Enterococcus* spp. in mechanically ventilated patients remains controversial, as these organisms are infrequently reported as primary causative agents of pneumonia [28].

Early- and late-onset ventilator-associated pneumonia are typically described as distinct entities, with different etiology and clinical outcomes, the latter being correlated with prolonged ICU and hospital LOS and increased in-hospital mortality rate [29]. Thus, consistent with these data, in our cohort the prevalence of late-onset VAP was higher, and patients experienced not only an increased ICU LOS, but also a longer duration of invasive mechanical ventilation.

In the present study, monomicrobial respiratory infections predominated, accounting for 85.5% (*n* = 53) out of all infections, while polymicrobial episodes were identified in 14.5% of cases (*n* = 9). This distribution is consistent with recent ICU studies which reported that monomicrobial etiology still remains the most frequent presentation of severe RTIs in patients admitted to ICU [26,30,31,32].

The antimicrobial resistance profile of the predominant Gram-negative pathogens identified in our cohort (*Acinetobacter baumannii*, *Pseudomonas aeruginosa* and *Klebsiella pneumoniae*) was marked by a high prevalence of advanced resistance phenotypes. Among all *A. baumannii* isolates, 66.7% strains (*n* = 18) were classified as either extensively drug-resistant or pandrug-resistant, whereas with respect to *P. aeruginosa*, 53.8% strains (*n* = 7) exhibited XDR or PDR profiles. Thus, the predominance of highly resistant Gram-negative pathogens observed in our study is in line with recent ICU-based studies which report an increase in XDR and carbapenem-resistant microorganisms causing HAP and VAP, particularly involving *A. baumannii* and *P. aeruginosa*, with a significant impact on patient outcome [33,34,35,36]. Overall, these findings highlight a declining antimicrobial susceptibility and ongoing treatment challenges with respect to severe respiratory infections in ICU settings.

Moreover, the predominance of carbapenem-resistant Gram-negative pathogens identified in our cohort is consistent with recent reports from neighboring countries. A systematic literature review focusing on Eastern European settings highlighted a significant prevalence of carbapenem-resistant *Enterobacterales* (CRE) and carbapenem-resistant *Pseudomonas aeruginosa* across several countries with epidemiological similarities to Romania, showing a regional burden of resistant Gram-negative infections [37]. Also, in line with our findings, a recent Bulgarian study focusing on ICU-admitted patients reported extensive carbapenem resistance among *Acinetobacter baumannii* isolates (100% resistance rates to imipenem and meropenem), with 21.9% of strains being classified as MDR and 78.1% as XDR, respectively [38]. Another ICU-based study from Serbia including mechanically ventilated patients also identified universal resistance rates of *Acinetobacter baumannii* strains to imipenem and meropenem, but preserved sensitivity to colistin, mirroring the results documented in our cohort [39].

Altogether, our findings are in line with reports from neighboring Eastern European countries documenting high resistance rates in ICU-related infections. However, compared to previously published regional studies which primarily focused on microbiological resistance patterns alone, our dataset integrates antimicrobial susceptibility profiling with modern organ dysfunction assessment, including SOFA-2 score. To our knowledge, this represents one of the first single-center ICU-based studies from Romania embracing a combined clinical-microbiological approach, providing additional insights into the interplay between pathogen resistance, host response and ICU-related outcomes in patients diagnosed with HAP and VAP. Moreover, large European multicenter analyses have demonstrated substantial variability in terms of pathogen distribution and resistance phenotypes across ICUs, highlighting the heterogeneity of resistance dynamics [40]. This variability underscores the importance of country-specific epidemiological surveillance, as regional data may not fully reflect center-specific resistance patterns.

We have also identified meaningful correlations between well-established clinical scores, including APACHE II, SOFA and SOFA-2 scores. Notably, the new development of SOFA-2 score in order to reflect daily organ support practices may offer additional value when integrated with traditional scoring systems in infection-related cohorts [14]. Thus, our findings provide preliminary evidence supporting the applicability of this recent score in critical respiratory infections, especially in future multicentric studies.

Acute kidney injury (AKI) was identified in 25.8% of patients and was associated with higher illness severity scores at ICU admission and at the time of infection diagnosis, as described in Supplementary Table S2. These findings are consistent with the recent literature showing that sepsis-associated AKI (SA-AKI) represents a frequent complication in critically ill patients with significant organ dysfunction burden [41,42]. Moreover, organ dysfunction-based scores may better reflect prognosis in AKI than admission severity scores alone, supporting our findings [43]. Although ICU mortality was higher in patients with AKI, this association did not reach statistical significance, in line with the variability reported in different ICU-cohort studies [44,45]. Within the scope of our study, AKI should therefore be interpreted primarily as a marker of global disease severity rather than as a direct consequence of specific antimicrobial resistance patterns.

The high mortality rate identified in our cohort is multifactorial and cannot be attributed solely to the virulence of the causative pathogens. Firstly, the high severity of acute illness, reflected by high APACHE II, SOFA and SOFA-2 scores, indicates a critically ill population with multiple organ dysfunctions. Secondly, an increased proportion of patients presented with pre-existing chronic organ insufficiencies (chronic heart failure—29%, chronic obstructive pulmonary disease—38.7%, cancer-related cachexia—12.9%, chronic kidney disease—6.4%), which limited physiological reserve and enhanced vulnerability to acute infectious insults. Thirdly, chronic conditions associated with immunosuppression (diabetes mellitus—16.1%, lung cancer—38.7%) further impaired host response mechanisms, contributing to adverse outcomes. Moreover, advanced age also represents a well-known risk factor for high mortality in ICU-acquired infections, probably due to the combined effects of immune senescence, multimorbidity and increased transition to chronic critical illness [46].

Thus, the reported mortality rate likely reflects the cumulative impact of MDR-related infections, acute disease severity, underlying comorbidities and critical-care severity.

Our study has several limitations. Firstly, its retrospective, single-center design may constrain the generalizability of the findings. Secondly, the relatively small sample size limited statistical power, particularly for subgroup analyses and exploratory correlations and reduced the feasibility of comprehensive multivariable modeling. Therefore, interpretation of these findings as representative epidemiological data on severe respiratory infections should be undertaken with caution.

Despite these limitations, our findings provide clinically relevant insights regarding the microbiological spectrum and its resistance patterns in severe respiratory infections diagnosed in ICU-admitted patients. One notable strength of this study is the integration of the recently updated SOFA-2 score into the clinical assessment of critical patients with severe respiratory infections. Another clinical aspect to be mentioned is represented by the local susceptibility profiles documented in this study, which may guide clinicians in tailoring empirical antibiotic treatment before the availability of susceptibility results, potentially improving early treatment adequacy in this vulnerable population. Taken together, the associations observed in the present study suggest that early risk stratification, integrated use of disease severity scores and timely antimicrobial stewardship may be critical in the management of critically ill patients with hospital-acquired or ventilator-associated pneumonia.

## 4. Materials and Methods

### 4.1. Study Design and Setting

This retrospective, observational and single-center study was conducted in the Intensive Care Unit of the Clinical Hospital of Pneumology of Iași, Romania, one of the region’s leading centers for the diagnosis and treatment of infectious and non-infectious respiratory diseases. All eligible patients were admitted to the ICU during a one-year period, from January to December 2025, respectively.

### 4.2. Study Population

All adult patients (≥18 years old) admitted to the ICU during the study period who had at least one positive endotracheal aspirate culture during ICU stay were retrospectively enrolled. Only patients with clinically significant respiratory infections were included in our study. Each patient was included only once in the analysis. Only the first positive endotracheal aspirate obtained from each patient was included in the analysis.

Patient inclusion criteria were:

✓ Adult patients (age ≥ 18 years old),
✓ Admission to ICU,
✓ Negative pharyngeal, nasal and rectal swabs at ICU admission,
✓ Positive endotracheal aspirate culture after 48 h of hospital (HAP) or ICU admission (VAP),
✓ Diagnosis of HAP or VAP.

Patient exclusion criteria were:

✓ Positive endotracheal aspirate culture obtained within the first 48 h of hospital admission (exclusion of community-acquired pneumonia),
✓ Positive endotracheal aspirate culture without clinical or biological signs of infection (considered as colonization),
✓ Duplicate isolates from the same infectious episode,
✓ Incomplete microbiological or clinical data,
✓ Death within the first 24 h after ICU admission.

### 4.3. Data Collection

Both clinical and laboratory data were retrospectively retrieved from ICU medical records and electronic databases and systematically recorded in a standardized Microsoft Excel sheet. All patient identifiers were removed prior to data analysis to ensure patient confidentiality.

The following variables were collected:

✓ Demographic data: age, gender, body mass index (BMI)
✓ Clinical Severity Scores: APACHE II score at ICU admission, SOFA and the recently updated version—SOFA-2—scores at the moment when respiratory tract infection was diagnosed
✓ Inflammatory biomarkers at ICU admission: NLR, PLR, C-reactive protein (CRP)
✓ ICU-related variables: length of stay (LOS), duration of invasive mechanical ventilation (expressed in hours of mechanical ventilation), ICU mortality rate
✓ Infection-related variables: type of respiratory infection (HAP, VAP), timing of endotracheal aspirate sampling during ICU stay
✓ Antimicrobial therapy: broad-spectrum (empirical) antibiotic treatment, duration of empirical therapy, targeted antimicrobial therapy according to susceptibility testing, need for antimicrobial therapy modification
✓ Microbiological outcomes: microbiological cure rate at seven days after implementing the targeted therapy.

### 4.4. Microbiological Analysis—Sampling, Bacterial Isolation and Antimicrobial Susceptibility Testing

Endotracheal aspirate samples were collected using standard sterile techniques and processed in the hospital microbiology laboratory in accordance with routine protocols.

Bacterial isolates were identified using a combination of standard microbiological techniques, including Gram staining and biochemical assays, together with automated identification performed on the Vitek 2 Compact system (BioMérieux, Marcy-l’ Etoile, France). Identification was carried out using Vitek 2 GP, Vitek 2 GN, Vitek 2 NH, Vitek 2 ANC and Vitek 2 YST identification cards, according to the manufacturer’s instructions and routine laboratory procedures. Antimicrobial susceptibility testing was performed using Vitek 2 automated system with Vitek 2 AST N440, Vitek 2 AST N437, Vitek 2 AST N439, Vitek 2 AST P659 and Vitek 2 AST ST03 cards. Interpretation of susceptibility results followed the European Committee on Antimicrobial Susceptibility Testing (EUCAST) standards, version 15.0 (2025), in order to ensure standardized and reproducible results. For all bacterial isolates, antimicrobial susceptibility testing was performed using automated methods, except for colistin, for which susceptibility was determined by broth microdilution method. Gram-negative and Gram-positive pathogens were analyzed separately, and microorganisms belonging to the ESKAPE group were also identified.

Isolates were subsequently classified as multidrug-resistant (MDR), extensively drug-resistant (XDR) and pandrug-resistant (PDR) according to the largely accepted definitions proposed by Magiorakos et al. in 2012 [47].

### 4.5. Definitions

Hospital-acquired pneumonia (HAP) was defined as pulmonary parenchymal infection occurring after at least 48 h of hospital admission, whereas ventilator-associated pneumonia (VAP) was considered when the infection of pulmonary parenchyma occurred in patients requiring invasive mechanical ventilation for more than 48 h.

VAP was classified as early-onset if it occurred in the first four days after the initiation of invasive mechanical ventilation and late-onset if it occurred after the fifth ventilator day, respectively.

The diagnosis of respiratory infection in all mechanically ventilated patients was based on the Clinical Pulmonary Infection Score (CPIS), which combines clinical parameters with biological and radiological findings, as follows: body temperature, peripheral white blood cell count, characteristics of tracheal secretions, oxygenation status based on the PaO_2_/FiO_2_ ratio, chest radiographic findings and positive microbiological results. Each variable was assigned a score between 0 and 2 points, resulting in a total CPIS ranging from 0 to 12 points. A total CPIS value of at least 6 points was considered suggestive of ventilator-associated pneumonia, in accordance with previously published criteria [48].

Empirical antimicrobial therapy was defined as broad-spectrum antibiotic treatment initiated before the availability of microbiological cultures and susceptibility results, while targeted therapy represents adjusted antibiotic treatment with respect to antibiogram results. Microbiological cure was defined as the absence of the causative pathogen in follow-up cultures seven days after adjusted antimicrobial therapy, when available.

Multidrug resistance was considered as non-susceptibility to at least one agent in three or more antimicrobial categories, extensively drug-resistance as non-susceptibility to at least one agent in all but two antimicrobial classes and pandrug resistance as non-susceptibility to all agents in all antimicrobial categories.

Incomplete microbiological data were defined as missing pathogen identification or inability to correlate microbiological findings with clinical infection. Thus, isolated absence of antimicrobial susceptibility testing did not constitute an exclusion criterion.

### 4.6. Statistical Analysis

Data analysis was performed using descriptive and exploratory methods. Continuous variables were assessed for distribution and are reported as median and interquartile range (IQR), as the variables did not follow a normal distribution. Categorical variables are reported as absolute numbers and percentages.

Group comparisons were performed using non-parametric tests, as appropriate. Continuous variables were compared between independent groups (multidrug-resistant versus non-multidrug-resistant infections, patients with versus without acute kidney injury) using the Mann–Whitney U test. Categorical variables were compared using the Chi-square test or Fisher’s exact test, based on expected cell counts.

Exploratory correlations between continuous variables were performed using Spearman’s rank correlation coefficient (*ρ*) and interpreted as follows: weak correlation if *ρ* < 0.30, moderate correlation if *ρ* = 0.30–0.59 and strong correlation if *ρ* ≥ 0.60.

Correlation analyses were performed on the entire study cohort, whereas comparative analyses between MDR and non-MDR infections excluded one isolate without standardized antimicrobial susceptibility testing.

All statistical tests were two-tailed and a *p*-value < 0.05 was considered statistically significant. Given the exploratory design and limited sample size, adjustments for multiple comparisons were not performed.

Statistical analysis was performed using IBM SPSS Statistics for Microsoft Windows, version 31 (IBM Corporation, Armonk, NY, USA).

### 4.7. Ethical Considerations

The study adhered to the Declaration of Helsinki. Given the retrospective nature of our study, patient consent was waived. Ethical approval for this study was provided by the Ethics Committee of the Clinical Hospital of Pneumology (No. 118/7 November 2024).

## 5. Conclusions

In this single-center observational study, severe respiratory infections diagnosed in critically ill patients admitted to an intensive care unit were predominantly monomicrobial and primarily caused by Gram-negative pathogens, with substantial antimicrobial resistance. Infections were mainly caused by agents belonging to the ESKAPE group. *Acinetobacter baumannii* and *Pseudomonas aeruginosa* accounted for the majority of infectious episodes and frequently exhibited multidrug-resistant, extensively drug-resistant and pandrug-resistant patterns.

Acute kidney injury emerged as a key marker of severe illness, showing strong associations with higher APACHE II, SOFA and SOFA-2 scores, along with a trend toward increased ICU mortality rate.

In settings with a high prevalence of multidrug-resistant pathogens, integrating clinical severity scores with epidemiological data may facilitate more accurate risk stratification and surveillance-guided empirical antimicrobial decisions.

Overall, these findings highlight the interplay between pathogen characteristics, host response and organ dysfunction in severe ICU respiratory infections. However, larger multicenter studies are needed to validate these findings and to better guide effective management strategies for multidrug-resistant respiratory infections in critically ill populations.

## Figures and Tables

**Table 1:** Baseline demographics and characteristics of the study population.

Variable	Value
**Age**, years, median (IQR)	71.5 (64.0–78.0)
**Male gender**, *n* (%)	41 (66.1)
**Body Mass Index (BMI)**, kg/m^2^, median (IQR)	24.95 (21.48–29.90)
**Diagnosis at ICU admission**, *n* (%)	
- Cardiorespiratory arrest	10 (16.1)
- Acute hypoxemic respiratory failure (type 1): acute respiratory distress syndrome (ARDS), acute pulmonary edema, hospital-acquired pneumonia	23 (37.1)
- Acute-on-chronic respiratory failure: chronic obstructive pulmonary disease (COPD), lung cancer	24 (38.7)
- Multiple Organ Dysfunction Syndrome (MODS)	3 (4.8)
- Hemorrhagic shock (massive hemoptysis)	2 (3.3)
**Comorbidities**, *n* (%) - Chronic ischemic heart disease - Hypertension - Chronic heart failure - Diabetes mellitus - Cancer-related cachexia - Severe anemia - Chronic kidney disease	7 (11.3) 21 (33.9) 18 (29) 10 (16.1) 8 (12.9) 4 (6.4) 4 (6.4)
**Type of respiratory tract infection**, *n* (%) - Hospital-acquired pneumonia - Ventilator-associated pneumonia Early-onset VAP Late-onset VAP	6 (9.7) 56 (90.3) 25 (44.6) 31 (55.4)
**ARDS at VAP diagnosis**, *n* (%)	24 (42.8)
**Patients requiring vasoactive agents**, *n* (%)	49 (79)
**APACHE II Score** at ICU admission, median (IQR)	23 (21–27)
**SOFA Score** at infection diagnosis, median (IQR)	8 (6–9)
**SOFA-2 Score** at infection diagnosis, median (IQR)	6.5 (5–8)
**Inflammatory biomarkers** at ICU admission, median (IQR)	11.5 (8–23) 237.5 (143–387) 9 (5.2–16.4)
- Neutrophil-to-lymphocyte ratio (NLR)	11.5 (8–23)
- Platelet-to-lymphocyte ratio (PLR)	237.5 (143–387)
- C-reactive protein (CRP), mg/dL	9 (5.2–16.4)
**ICU length of stay (LOS)**, days, median (IQR)	11 (6–18)
**Duration of invasive mechanical ventilation**, hours, median (IQR)	246 (144–384)
**ICU mortality rate**, *n* (%)	51 (82.3)

Note: Continuous variables are expressed as median (interquartile range), while categorical variables are reported as absolute numbers and percentages.

**Table 2:** Antimicrobial resistance classification of bacterial isolates identified in endotracheal aspirates.

Microorganism	Isolates Number, *n*	NonMDR, *n*	MDR, *n*	XDR, *n*	PDR, *n*
*A. baumannii*	27	3	6	15	3
*P. aeruginosa*	13	2	4	6	1
*K. pneumoniae*	4	1	0	1	2
*C. striatum*	6	1	5	0	0
*S. pneumoniae*	4	3	1	0	0
*E. coli*	2	1	1	0	0
*S. maltophilia*	1	0	1	0	0
*P. vulgaris*	1	0	0	1	0
*S. marcescens*	1	0	1	0	0
MRSA	2	0	2	0	0
MSSA	1	1	0	0	0
*H. influenzae*	2	2	0	0	0
*E. durans*	1	1	0	0	0
*K. rosea*	1	NA *	NA *	NA *	NA *
Total number of isolates	66	15	21	23	6

Note: MDR, XDR and PDR status were recorded as independent variables for each bacterial isolate, reflecting the original microbiological classification. * NA—Not available. For *Kocuria rosea*, antimicrobial susceptibility testing could not be interpreted due to the absence of EUCAST standardized breakpoints; thus, resistance classification was not performed.

**Table 3:** Characteristics of empirical and targeted antimicrobial therapy in critically ill patients with HAP/VAP.

Parameter	Value
**Empirical antibiotic regimen** 1. Carbapenem-based regimen, *n* (%) 1.1 monotherapy, *n* (%) 1.2 combinations, *n* (%) 2. β-lactam/β-lactamase inhibitor, *n* (%) 2.1 monotherapy, *n* (%) 2.2 combinations, *n* (%) 3. Third-generation cephalosporin-based regimen, *n* (%) 3.1 monotherapy, *n* (%) 3.2 combinations, *n* (%) 4. Other regimens, *n* (%)	17 (27.4) 8 (12.9) 9 (14.5) 13 (21) 9 (14.5) 4 (6.5) 20 (32.2) 11 (17.7) 9 (14.5) 12 (19.4)
**Duration of empirical antibiotic treatment** (days), median (IQR)	3.0 (2.0–5.0)
**Duration of targeted therapy (days), median** (IQR)	6.0 (3.0–10.0)
**Total duration of antibiotic therapy (days)**, median (IQR)	9.0 (6.0–14.0)
**Microbiological cure rate 7 days from targeted therapy initiation**, *n* (%)	27.0 (43.5)

**Table 4:** Comparison between MDR and non-MDR infections.

Variable	MDR Infections (*n* = 50), Median (IQR)	Non-MDR Infections (*n* = 11), Median (IQR)	*p*-Value
Age, years, median (IQR)	71.5 (65.0–79.0)	69.0 (60.5–77.0)	0.814 *
APACHE II score, median (IQR)	23.0 (21.0–27.0)	21.0 (20.0–23.0)	0.086 *
SOFA score, median (IQR)	8.0 (6.0–10.0)	7 (6.0–8.5)	0.610 *
SOFA-2 score, median (IQR)	7.0 (6.0–8.0)	5.0 (5.0–6.5)	0.153 *
NLR, median (IQR)	12.0 (8.00–24.00)	8.0 (5.5–18.5)	0.094 *
PLR, median (IQR)	242.0 (128.0–387.0)	184.0 (164.0–351.5)	0.881 *
CRP, mg/dL, median (IQR)	9.05 (5.46–17.98)	6.45 (1.95–11.77)	0.099 *
ICU LOS, days, median (IQR)	10.5 (6.0–17.0)	11.0 (8.0–20.5)	0.403 *
Duration of invasive mechanical ventilation, hours, median (IQR)	216.0 (120.0–372.0)	288.0 (243.5–636.0)	0.062 *
AKI, *n* (%)	13.0 (81.3)	3.0 (18.7)	1.000 **
Mortality rate, *n* (%)	39.0 (78.0)	11.0 (100.0)	0.188 **

* Mann–Whitney U test. ** Fisher’s exact test.

**Table 5:** Comparison of monomicrobial and polymicrobial respiratory infections.

Variable	Monomicrobial Infections (*n* = 53)	Polymicrobial Infections (*n* = 9)	*p*-Value
Age, years, median (IQR)	72.0 (65.0–81.0)	63.0 (46.5–77.0)	0.066 *
APACHE II score, median (IQR)	23.0 (21.0–25.0)	23.0 (21.0–27.0)	0.904 *
SOFA-2 score, median (IQR)	7.0 (5.0–8.0)	6.0 (5.0–7.0)	0.369 *
SOFA, median (IQR)	8.0 (6.0–10.0)	8.0 (7.0–9.0)	0.519 *
AKI development, *n* (%)	15.0 (28.3)	1.0 (11.1)	0.425 **
ICU LOS, days, median (IQR)	9.0 (6.0–17.0)	16.0 (12.0–30.0)	0.061 *
Hospital LOS, days, median (IQR)	14.0 (7.0–23.0)	26 (16.0–32.0)	**0.044** *
Duration of MV, hours, median (IQR)	235.0 (144.0–360.0)	372.0 (180.0–432.0)	0.280 *

* Mann–Whitney U test. ** Fisher’s exact test.

## Data Availability

The data presented in this study are available upon request from the corresponding author due to privacy reasons.

## Supplementary Materials

The following supporting information can be downloaded at: https://www.mdpi.com/article/10.3390/antibiotics15020232/s1, Supplementary Table S1: Distribution of major bacterial categories in early- *versus* late-onset VAP; Supplementary Table S2: Associations between acute kidney injury and disease severity scores and ICU-related outcomes; Supplementary Table S3: Correlations between severity scores, inflammatory biomarkers and ICU-related outcomes.

## Abbreviations

The following abbreviations are used in this manuscript:

ICUs	Intensive Care Units;
HAP	Hospital-Acquired Pneumonia;
VAP	Ventilator-Associated Pneumonia;
CAP	Community-acquired pneumonia;
LOS	Length of Stay;
LRTIs	Lower Respiratory Tract Infections;
RTIs	Respiratory Tract Infections;
MDR	Multidrug-resistant;
AMR	Antimicrobial Resistance;
APACHE II	Acute Physiology and Chronic Health Evaluation II;
SOFA	Sequential Organ Failure Assessment;
SOFA-2	Sequential Organ Failure Assessment 2;
NLR	Neutrophil-to-Lymphocyte Ratio;
PLR	Platelet-to-Lymphocyte Ratio;
BMI	Body Mass Index;
CRP	C-reactive protein;
EUCAST	European Committee on Antimicrobial Susceptibility Testing;
MRSA	Methicillin-resistant *Staphylococcus aureus*;
MSSA	Methicillin-susceptible *Staphylococcus aureus*;
XDR	Extensively drug-resistant;
PDR	Pandrug-resistant;
AKI	Acute kidney injury;
SA-AKI	Sepsis-associated acute kidney injury;
MODS	Multiple Organ Dysfunction Syndrome;
ARDS	Acute Respiratory Distress Syndrome;
COPD	Chronic Obstructive Pulmonary Disease;
CPIS	Clinical Pulmonary Infection Score;
CRE	Carbapenem-resistant *Enterobacterales*
