# Supplementary material for: Clinical and Microbiological Profile of Hospital-Acquired and Ventilator-Associated Pneumonia in Critically Ill Patients: A Retrospective Observational Study
Source: Antibiotics (Basel). 2026 Feb 22;15(2):232. doi: 10.3390/antibiotics15020232 (PMC12938439; doi:10.3390/antibiotics15020232)
Supplement: Supplementary file 1 [file antibiotics-15-00232-s001.zip › antibiotics-4128192-supplementary.pdf]

Supplementary Files

Clinical and Microbiological Profile of Hospital-Acquired and Ventilator-Associated Pneumonia in Critically Ill Patients: A Retrospective Observational Study

1.

Supplementary Table S1. Distribution of major bacterial categories in early- *versus* late-onset VAP

**Bacterial category**	**Early-onset VAP (*n* = 25)**	**Late-onset VAP (*n* = 31)**	***p-Value***
**Non-fermenting Gram-negative bacteria** (*Acinetobacter baumannii, Pseudomonas aeruginosa, Stenotrophomonas maltophilia*), *n*(%)	17 (48.6)	18 (51.4)	0.580
**Fermenting Gram-negative bacteria** (*Haemophilus influenzae*), *n*(%)	0 (0)	1 (100)	1
Enterobacterales (*Klebsiella pneumoniae, Escherichia coli, Serratia marcescens, Proteus vulgaris*), *n*(%)	1 (12.5)	7 (87.5)	0.063
**Gram-positive bacteria** (*Corynebacterium striatum, Staphylococcus aureus, Streptococcus pneumoniae*), *n*(%)	6 (46.2)	7 (53.8)	1

Note: Bacterial categories were not mutually exclusive due to the presence of polymicrobial infections. Categorical variables were compared using chi-square test or Fisher's exact test, as appropriate.

2.

Supplementary Table S2. Associations between acute kidney injury and disease severity scores and ICU-related outcomes

**Parameter**	**AKI (*n* = 16), median (IQR)**	**Non-AKI (*n* = 46), median (IQR)**	***p-Value***
APACHE II score	26.0 (23.0 – 32.0)	22.5 (20.0 - 24.0)	**0.002***
SOFA score	10.0 (8.50 - 12.0)	7.0 (6.0 - 9.0)	**< 0.001***
SOFA-2 score	8.5 (6.50 – 11.50)	6.0 (5.0 – 7.0)	**0.003***
ICU mortality, *n* (%)	16 (100)	35 (76.1)	0.052**
ICU LOS, days	8.0 (6.5 – 23.5)	12.0 (6.0 – 18.0)	0.961*
Duration of invasive mechanical ventilation, hours	225.5 (146.0 -408.0)	269.0 (132.0 -384.0)	0.853*

*Mann-Whitney U test

**Fisher's exact test

3.

Supplementary Table S3. Correlations between severity scores, inflammatory biomarkers and ICU-related outcomes

**Correlated variables**	**Spearman's rho (*ρ*) value**	***p-Value***
APACHE II score ↔ SOFA score	0.34	0.006
APACHE II score ↔ SOFA-2 score	0.29	0.023
SOFA-2 score ↔ duration of invasive mechanical ventilation	-0.26	0.037

Note: Correlations between continuous variables were assessed by using Spearman's rank correlation coefficient.
